# Perceptual and memory functions in a cortex-inspired attractor network model

**DOI:** 10.1186/1471-2202-12-S1-K2

**Published:** 2011-07-18

**Authors:** Anders Lansner

**Affiliations:** 1 Dept of Computational Biology, School of Computer Science and Communication, Stockholm University and Royal Institute of Technology, Stockholm Brain Institute partner, Sweden

## 

I will initially present the foundations of this work in Hebb’s hypotheses of synaptic plasticity and cell assemblies and how perception and memory can be understood in those terms. Then I will turn to the question about biological plausibility, which we have studied by means of modeling and computer simulation, typically using very-large scale Hodgkin-Huxley based network models running on supercomputers. We developed and studied a computational model of cortical layers 2 and 3 where the horizontal connectivity critical in this context is most prominent. Sparse, distributed memory items were embedded in such a network by means of Hebbian synaptic plasticity. I will show how this model can perform basic perceptual and memory functions like perceptual completion and rivalry as well as reconstructive associative recall from fragments of a stored memory. It also reproduces key features of the attentional blink phenomenon. More recently we analyzed the dynamics of these processes in terms of spike discharge patterns, oscillatory dynamics in theta to gamma frequencies, as well as spontaneous ongoing activity, phase locking and coherence. It was shown that some of the dynamic properties depend prominently on the modular structure of the network. It is concluded that a network model set up along the lines proposed by Hebb with cortex-like composition, microcircuit structure, and connectivity can to a significant extent reproduce basic cognitive functions as well as dynamics of real cortex. Finally, directions for further development of this type of models will be discussed, e.g. to use them as components in network-of-network architectures representing complex interacting cortical feedforward, lateral and processing streams.

